# Comparative metabolomic profiling of *Lupinus albus* and *L. angustifolius* harvest residues: exploring chemical diversity and valorization potential

**DOI:** 10.3389/fpls.2025.1617634

**Published:** 2025-07-07

**Authors:** Salma Halime, Jenny Renaut, Stéphanie Zimmer, Hanna Heidt, Cédric Jacquard, Kjell Sergeant

**Affiliations:** ^1^ GreenTech Innovation Centre, Luxembourg Institute of Science and Technology, Hautcharage, Luxembourg; ^2^ Université de Reims Champagne-Ardenne, INRAE, Unité de Recherche RIBP USC, Reims, France; ^3^ Research and Development, Institute for Organic Agriculture and Agroecology Luxembourg a.s.b.l. (IBLA), Medernach, Luxembourg

**Keywords:** *L. albus*, *L. angustifolius*, harvest residues, saponins, flavonoids, valorization

## Abstract

Lupin species are a rich source of bioactive compounds with diverse industrial applications, yet their harvest residues remain underutilized. This study investigates the metabolomic composition of the harvest residues of different *Lupinus albus* and *L. angustifolius* varieties to explore species-specific biochemical differences and valorization potential. Methanolic extracts from the harvest residues were analyzed using UHPLC-MS/MS, leading to the tentative identification of 181 compounds, with saponins and flavonoids identified as the predominant metabolite classes. The data reveal distinct metabolic profiles: *L. albus* is characterized by higher levels of isoflavonoids (luteone), flavonols (isorhamnetin), and flavanones (naringenin), which were detected as free aglycones, glycosylated derivatives, and, for some compounds, as malonylated glycosides, which correlate with a higher antioxidant capacity. In contrast, extracts from *L. angustifolius* contain higher amounts of saponins, particularly soyasaponins B, E, A, and DDMP, as well as flavones (chrysoeriol and derivatives) and isoflavones (genistein and wighteone and its derivatives). Correlation analysis confirmed that a positive relationship exists between flavonoids and antioxidant activity, while saponins showed a negative correlation with antioxidant potential. This study highlights the distinct valorization opportunities of *Lupinus* residues: *L. albus*, rich in antioxidants and defense-related metabolites, holds promise for nutraceutical, pharmaceutical, and functional food applications as well as plant protection strategies. In contrast, *L. angustifolius*, with its high saponin content, has potential for biopesticides and antimicrobial agents. This study underscores the sustainability potential of *Lupinus* harvest residues as a renewable resource, supporting the upcycling of agricultural byproducts into high-value bioactive products.

## 1 Introduction

Sustainability has become a cornerstone of global development, driving the quest for innovative strategies to address environmental challenges while fostering economic growth. A key approach to achieving these goals is the circular economy, a transformative framework prioritizing efficient resource utilization, waste reduction, and material repurposing (Oliveira et al., 2021). Within this framework, agriculture plays a pivotal role by not only producing renewable biological resources but also adopting practices that improve nutrient cycling, soil regeneration, and bio-waste valorization (Xu et al., 2022). Among the most significant yet underutilized by-products of agriculture are agro-residues, generated in vast quantities during crop harvesting. With global production estimated at 3.8 billion Mg annually (Puglia et al., 2021), these residues offer considerable potential for sustainable resource utilization. However, common practices involve ploughing in the soil as green manure, burning, or landfilling harvest residues, leading to serious environmental consequences, including greenhouse gas emissions, particulate matter pollution, and soil and water degradation (Phiri et al., 2024).

Recent advancements have revolutionized the utilization of agricultural residues, moving from traditional practices to innovative applications such as biofuel and bioenergy production. This transformation is driven not only by technological advancements but also by global policy frameworks such as the Sustainable Development Goals (SDGs), which promote sustainable production, low-carbon development, and the transition to renewable resources as part of a broader commitment to environmental and socio-economic resilience (Paudel et al., 2024). Beyond their utilization for energy production, a down-cycling approach, field residues such as rice straw, wheat straw, corn stalks, and sugarcane leaves also have an upcycling potential as sources of bioactive compounds. For example, rice straw is a rich source of cellulose fractions and phenolic compounds which antioxidant properties can generate value in food and nutrition (Ramos et al., 2023). Sugarcane leaves have a high content of ferulic and p-coumaric acids, which exhibit recognized anti-inflammatory and antimicrobial properties (Hewawansa et al., 2024). Similarly, corn stover, rich in lignocellulosic materials, is gaining attention as it can be used as feedstock for the production of bio-based materials and biochemicals (da Rosa et al., 2025).

The increasing societal demand for non-animal-based protein sources for both human and animal nutrition has driven the expanded cultivation of *Fabaceae* species (Shavanov, 2021). While fodder crops such as *Medicago sativa* and *Trifolium* spp. are harvested and directly utilized as animal feed without generating significant residues (Sowiński and Adamczewska-Sowińska, 2022), species cultivated for their grains or pods (e.g., *Glycine max*, *Phaseolus vulgaris*, *Pisum sativum)* and used for human consumption result in the generation of harvest residues in the form of vines and pods. For some of the latter crops, the residues are currently already being studied for the valorization potential due to their rich content of bioactive metabolites (Dimopoulou et al., 2024; Shea et al., 2024). Soybean by-products are abundant in isoflavones and saponins, which exhibit antioxidant, anti-inflammatory, and anticancer properties, making them highly valuable for nutraceuticals and cosmetics (Oleszek et al., 2023). Similarly, faba bean pods contain phenolics such as catechin and syringic acid, which have applications in food and pharmaceuticals (Krenz et al., 2024). Pea vine residues have also been valorized for their sugars, phenolics, and biopolymers, offering sustainable opportunities in material and bioenergy production (Xia et al., 2016).

Lupins (*Lupinus* spp.), another genus of the *Fabaceae* family, have attracted growing attention for their contributions to sustainable agriculture. Cultivated in Europe, Australia, and Mediterranean regions, lupins serve a dual purpose: producing protein-rich seeds for human and animal nutrition while improving soil health through biological nitrogen fixation (Lucas et al., 2015). With seeds containing up to 44% protein, lupins represent an excellent alternative protein source for global food and feed systems (Chukwuejim et al., 2024; Ishaq et al., 2022). In the European Union, lupin cultivation has expanded significantly, growing from 222,220 hectares in 2020 to 391,342 hectares in 2021, contributing 28.7% of global lupin production (Ačko et al., 2023; Maia et al., 2023). In addition to seed production, lupin farming generates considerable biomass residues, approximately 7 tons of residues per ton of seeds harvested (Heredia Salgado et al., 2022). These residues, consisting of stalks, stubbles, pod shells, and husks, are commonly managed through field incorporation, or soil amendment to increase organic matter, nitrogen, phosphorus, and microbial diversity (Hansen et al., 2021; Zheng et al., 2022). While these practices contribute to improved soil fertility, the potential of lupin harvest residues for high-value applications remains largely untapped. Recent research highlights the feasibility of valorizing lupin biomass as an alternative feed source for ruminants. With superior crude protein and metabolizable energy content compared to cereal straws, lupin residues have been shown to meet up to 85% of energy and 50% of protein requirements for sheep, offering a sustainable and innovative approach within the framework of the circular economy (Maia et al., 2023). Additionally, agro-residues from lupin have been explored as feedstock for biochar production, which can further enhance soil quality and sequester carbon. For example, Salgado et al., 2018 reported theoretical biochar yields of 28.4 wt% from lupin residues in South America, demonstrating the growing interest in diverse and sustainable valorization pathways for these by-products.

Among lupin species, *Lupinus albus* (white lupin) and *L. angustifolius* (blue or narrow-leafed lupin) are of particular interest due to their adaptability and contribution to sustainable farming systems (Ishaq et al., 2022). *L. albus* thrives in nutrient-poor Mediterranean soils, while *L. angustifolius* is more drought tolerant and better adapted to cooler climates (Gataulina et al., 2021; Gresta et al., 2017). Beyond their agronomic traits, these species are notable for their rich profiles of secondary metabolites, particularly saponins and flavonoids (Andersen et al., 2022; Ferchichi et al., 2021; Siger et al., 2023).

Saponins are amphipathic glycosides composed of hydrophilic glycone moieties linked to hydrophobic aglycones (sapogenins) via ether or ester glycosidic bonds (Singh et al., 2017; Woldemichael et al., 2003). Based on the structure of their aglycone, saponins are classified into two primary groups: triterpenoid and steroidal saponins (Le Bot et al., 2022). The attached sugar units typically include hexoses (glucose, galactose), 6-deoxyhexoses (rhamnose, furanose), pentoses (arabinose, xylose), and uronic acids (glucuronic acid, galacturonic acid) (Pollier et al., 2011). Triterpene aglycones can undergo various functional group substitutions, contributing to the structural diversity of saponins. In legumes, saponins are further categorized into primary groups such as Group A, Group B, Group E, and DDMP saponins (Singh et al., 2017). Saponins play ecological roles in plant defense and exhibit antimicrobial, anti-inflammatory, and hemolytic properties, making them valuable for industrial and pharmaceutical applications (Kareem et al., 2022; Rai et al., 2021). Similarly, flavonoids, characterized by their C6–C3–C6 backbone structure, are major phenolic compounds in legumes. They are categorized into subgroups such as anthocyanins, flavones, isoflavones, and flavanols (Dias et al., 2021; Serventi and Dsouza, 2020). These compounds play crucial roles in plant defense, UV protection, and pigmentation while also offering significant health benefits due to their antioxidant, anti-inflammatory, and anticancer properties. Flavonoids contribute to the scavenging of free radicals, modulation of enzyme activity, and regulation of cell signaling pathways (Baskar et al., 2018).

The agricultural significance of *L. albus* and *L. angustifolius*, combined with the bioactive potential of their secondary metabolites, has led to extensive research into their metabolomic profiles. Previous studies have primarily focused on seeds (Caramona et al., 2024; Hellal et al., 2021; Liu and Jiang, 2022), aerial parts (Andersen et al., 2022; Wojakowska et al., 2013), and roots (Frémont et al., 2022), revealing the remarkable diversity and bioactive potential of these metabolites. Additionally, species-specific differences in lipid, alkaloid, and flavonoid profiles have been identified (Czubinski et al., 2021; Ferchichi et al., 2021; Valente et al., 2024), underscoring the chemical diversity of these species. Recently, research has also focused on the residues remaining after grain harvesting, such as straws and pod shells, with a particular focus on alkaloid profiling. These studies have highlighted the potential of these harvest residues as sustainable feed for ruminant animals (Maia et al., 2023). All these studies, whether focused on seeds, aerial parts, or harvested residues, have highlighted the significant potential of these two species. However, a knowledge gap persists in the characterization of the metabolomic profile of the biomass residues remaining after grain harvesting. Despite their abundance, these residues remain vastly underutilized, with limited knowledge of their metabolomic composition and potential applications. This study addresses this gap by comparing the metabolite profiles of biomass residues from *L. albus* and *L. angustifolius*. Hypothesizing that the different genetic backgrounds of these species will result in differences in the extracted metabolome, the metabolomic profile of eight cultivars of lupins, five *L. angustifolius* and three *L. albus*, were compared, thereby highlighting differences between the species but between varieties within each species. To our knowledge, this is the first study that characterizes the metabolic profile of harvest residues of lupins in an untargeted way, thereby opening the possibility of a valorization of this overlooked biomass and its use as feedstock in the development of a circular economy.

## 2 Materials and methods

### 2.1 Chemicals and reagents

The chemicals utilized in this study were sourced from commercial suppliers, including methanol (Sigma Aldrich, 34860), formic acid (LGC Standards-Promochem, SO-9679-B001), 2,4,6-Tri(2-pyridyl)-s-triazine (Sigma Aldrich, T1253), acetonitrile (Carl Roth, HN40.2), iron(II) sulfate heptahydrate (FeSO_4_·7H_2_O) (VWR, 24,244.232), iron(III) chloride hexahydrate (FeCl_3_·6H_2_O) (Merck VWR, 1.03943250), hydrochloric acid (HCl) 0.1 M (ChemLab, 31955373), sodium acetate trihydrate (C_2_H_3_NaO_2_·3H_2_O) (Sigma Aldrich, 331058-100G), and glacial acetic acid (CH_3_COOH) (J.T. Baker, 9524-33).

### 2.2 Plant materials

This study analyzed harvest residues from eight varieties of Lupinus spp., consisting of five blue lupin (*L. angustifolius*) varieties (Jowisz, Bolero, Boregine, Lunabor, and Probor) and three white lupin (*L. albus*) varieties (Celina, Frieda, and Dieta). The samples were collected after harvest of the official lupin variety trials in organic farming for Luxembourg by the Institute for Organic Agriculture and Agroecology Luxembourg a.s.b.l. (IBLA), where the lupins were sown in April 2023. The blue lupins were harvested in August 2023, and the white lupins in September 2023 in Hupperdange canton Clervaux. Data of each variety were collected during the vegetation period, as well as soil and weather data. Upon arrival at LIST, the dried harvest residues were ground into a fine powder using a Retsch Cutting Mill SM 300. The powdered samples were subsequently vacuum-packed in airtight bags and stored at 4°C for further analysis.

### 2.3 Metabolite extraction

Fifty mg of grounded harvest residues were weighed. The extraction was performed with 80% MeOH/20% MQ (Milli-Q water, Merck Millipore, Darmstadt, Germany) at a ratio of 1:12 (w/v). The solvent was added to the samples and shaken using a Thermomixer (Eppendorf, Germany) for four hours at room temperature. Subsequently, the mixtures were centrifuged at 14000 rpm for 25 min at 4°C, and supernatants were collected and stored at -20°C prior to analysis.

### 2.4 Untargeted metabolomics analysis with UHPLC-MS/MS and identification

The extracts, initially extracted in 80% MeOH, were diluted with Milli-Q water to attain a final concentration of 20% MeOH/MQ. The diluted extracts were filtered through a 0.22 μm PTFE syringe filter (Millex-LG, Merck KGaA, Darmstadt, Germany) and analyzed using an Acquity UPLC I-Class ultra-high-pressure liquid chromatography (UHPLC) system equipped with a diode array detector (DAD) (Waters, Milford, MA, USA) coupled to a hybrid quadrupole-time of flight mass spectrometer (TripleTOF 6600+, SCIEX, Framingham, MA, USA) in both positive and negative ionization modes, as previously described (Backes et al., 2021). Ten microliters of the sample were injected and separated on a reverse-phase Acquity UPLC BEH C18 column (2.1 × 100 mm, 1.7 μm particle size) (Waters, Milford, MA, USA) at a flow rate of 0.5 mL/min and a column temperature of 50°C. The mobile phase consisted of 0.1% (v/v) formic acid in water (A) and 0.1% (v/v) formic acid in acetonitrile (B), with the following gradient: 0 min, 1% B; 4 min, 1% B; 16 min, 5% B; 35 min, 40% B; 45 min, 100% B; 50 min, 100% B; 54 min, 1% B; and 60 min, 1% B. UV–visible spectra were acquired between 190 and 800 nm at a rate of 10 points/sec.

Electrospray ionization (ESI) was performed on analytes using the following parameter values for positive and negative modes: source temperature 650°C; ion spray voltage of 4.5 and -4.5 kV, respectively, curtain gas (nitrogen) of 30 psi, nebulizer gas (air) of 55 psi and turbine gas (air) of 50 psi. The declustering potential was set up at 60 V in positive and -60 V in negative mode. Survey scans of 175 ms were acquired for information-dependent acquisition. The ten highest MS ions were selected for fragmentation if they were singly charged and had an intensity exceeding a threshold of 100 counts/sec. Product ion scans were collected with an accumulation time of 200 ms in high sensitivity mode, thus leading to a total cycle time of 2.225 s. A sweeping collision energy of 15 V below and above 15 and -15 V, for the positive and negative modes, respectively, was applied to all precursor ions. The dynamic exclusion was set for 2 s after three occurrences before the precursor could be fragmented again. Data for all varieties were acquired in negative mode, while positive mode analysis was run on one variety of each species to aid in the identification of the compounds.

For the identification and relative quantification, raw data files were processed using Progenesis QI (v2.3, Nonlinear Dynamics, Newcastle upon Tyne, UK) to align all runs, normalize the data, and perform relative quantitative analysis based on sample groups (species). Only features with MS/MS data were retained for the identification stage. The output data were manually reviewed using PeakView software (v1.2, SCIEX, Framingham, MA). Initial identification relied on the use of an in-house database exported in msp-format containing mainly saponins previously identified in seven Fabaceae species, including *L. angustifolius* and *L. albus*. All database hits were manually validated. For compounds not present in this in-house database, identification was achieved through a literature search, for instance the elaborate flavonoid catalogue identified in Mexican lupin described by (Stobiecki et al., 2010), and MS/MS comparison using external databases such as GNPS (https://gnps.ucsd.edu/ProteoSAFe/libraries.jsp), MZCloud™ (https://beta.mzcloud.org/), LipidMaps (https://www.lipidmaps.org/), and PubChem (https://pubchem.ncbi.nlm.nih.gov). Accepted identifications were subsequently incorporated into the in-house database for future dereplication. All identifications reported in this study adhere to level 2 standards as defined by the Metabolomics Standards Initiative (MSI).

### 2.5 Ferric reducing/antioxidant power assay

The FRAP assay measures antioxidant capacity based on the reducing power of molecules to convert Fe(III) to Fe(II) (Benzie and Strain, 1996). For the assay, 10 μl of extracted samples were mixed with 190 μl of freshly prepared FRAP reagent (300 mM acetate buffer, 10 mM ferric-tripyridyltriazine, 20 mM FeCl3; pH 3.6) in a 96-well plate. The plate was covered with aluminum foil and incubated at room temperature for 20 minutes. A standard curve was prepared using FeSO4 at varying concentrations (0, 0.05, 0.1, 0.2, 0.4, 0.6, 0.8, and 1 mM), yielding the equation y=0.6787x+0.077y (R^2^ = 0.9981). Antioxidant capacity was expressed as mM ferrous equivalents. Absorbance was measured at 595 nm using a spectrophotometric microplate reader (Spark, Tecan).

### 2.6 Statistical analysis

The data were analyzed to assess differences between the two lupin species and within each species. Statistical analysis of saponin and flavonoid contents was performed using Progenesis, where features with a p-value < 0.05 were selected based on an internally applied one-way ANOVA. To further explore variation in flavonoid and saponin contents across the samples, Partial Least Squares Discriminant Analysis (PLS-DA) and hierarchical clustering heatmaps were generated using MetaboAnalyst 6.0 (www.metaboanalyst.ca). Both PLS-DA and heatmap data were normalized by median and subjected to Pareto scaling and square root transformation to enhance the representation of the underlying data structure. The correlation analysis was conducted using the Spearman rank correlation test on normalized metabolite data to evaluate the relationship between metabolite abundance and antioxidant capacity using OriginPro 2024. Antioxidant capacity was analyzed separately using one-way ANOVA followed by Tukey’s *post hoc* test (p < 0.05) in OriginPro 2024.

## 3 Results

### 3.1 Untargeted metabolomic profile of the two species of lupins*: L. albus* (white lupin) and *L. angustifolius* (blue lupin)

**Figure 1:**
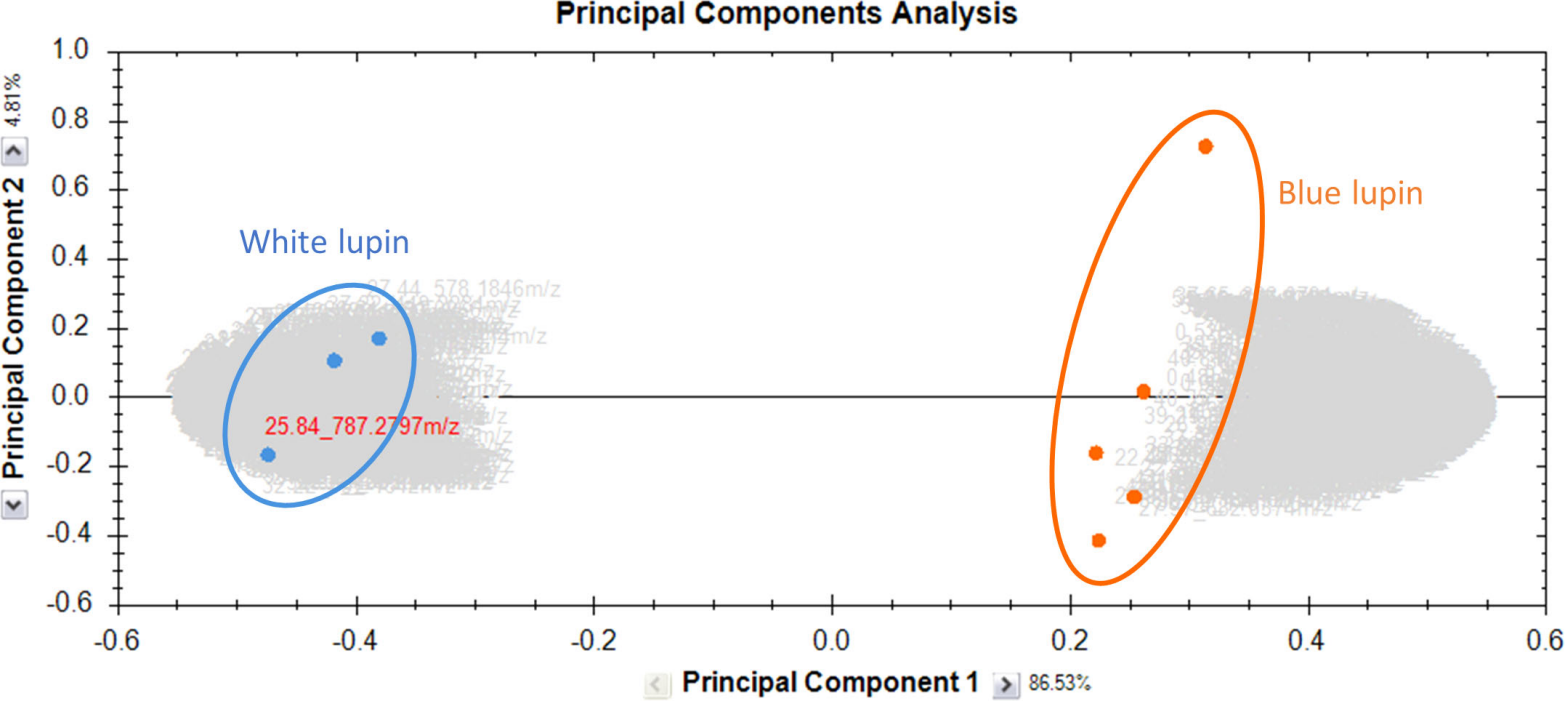
PCA of the metabolomic profiles of methanolic extracts from the harvest residues of two *Lupinus* species. Orange dots represent the blue varieties, while blue dots represent the white varieties. The first principal component (PC1) explains 86.53% of the total variance, while the second principal component (PC2) accounts for 4.81%. This graph was generated using Progenesis QI.

The methanolic extracts of harvest residues from two *Lupinus* species were analyzed using UHPLC-MS/MS. Data processing was performed using Progenesis QI (v2.3, Nonlinear Dynamics, Waters, Newcastle, UK). Principal component analysis (PCA) was first applied to visualize sample clustering and assess variability, revealing a distinct metabolic differentiation between the two species. PC1 explained 86.53% of the variance, while PC2 accounted for 4.81%, highlighting species-specific metabolic differences as the primary source of variation (**Figure 1**). Subsequently, features were filtered based on statistical significance (fold change >2, p-value <0.05) to retain only those with substantial differences in abundance. The selected features were subsequently identified through MS/MS fragmentation analysis and databases matching, leading to the annotation of 181 compounds covering diverse phytochemical classes. Among these, saponins and flavonoids were the predominant metabolite classes identified in both lupin species.

#### 3.1.1 Identification of saponins

The saponins identified in this study all possess an oleanane-type aglycone. In both species analyzed, the saponins contain a triterpenic pentacyclic nucleus belonging to the β-amyrin class. The β-amyrin skeleton can undergo further modifications through oxidative reactions mediated by cytochrome P450 enzymes. These modifications result in the formation of structurally diverse saponins, characterized by the presence of hydroxyl or carboxyl groups at specific positions on the triterpenic backbone (Tava et al., 2011). The identified saponins contain either one or two sugar moieties. In the nomenclature, the symbol (x) indicates the presence of two sugar chains. When sugars are enclosed in parentheses, it signifies that they are branched. The sugar residues are classified as follows: hexoses (Hex), pentoses (Pent), uronic acids (HexA), and deoxyhexoses (dHex).

**Figure 2:**
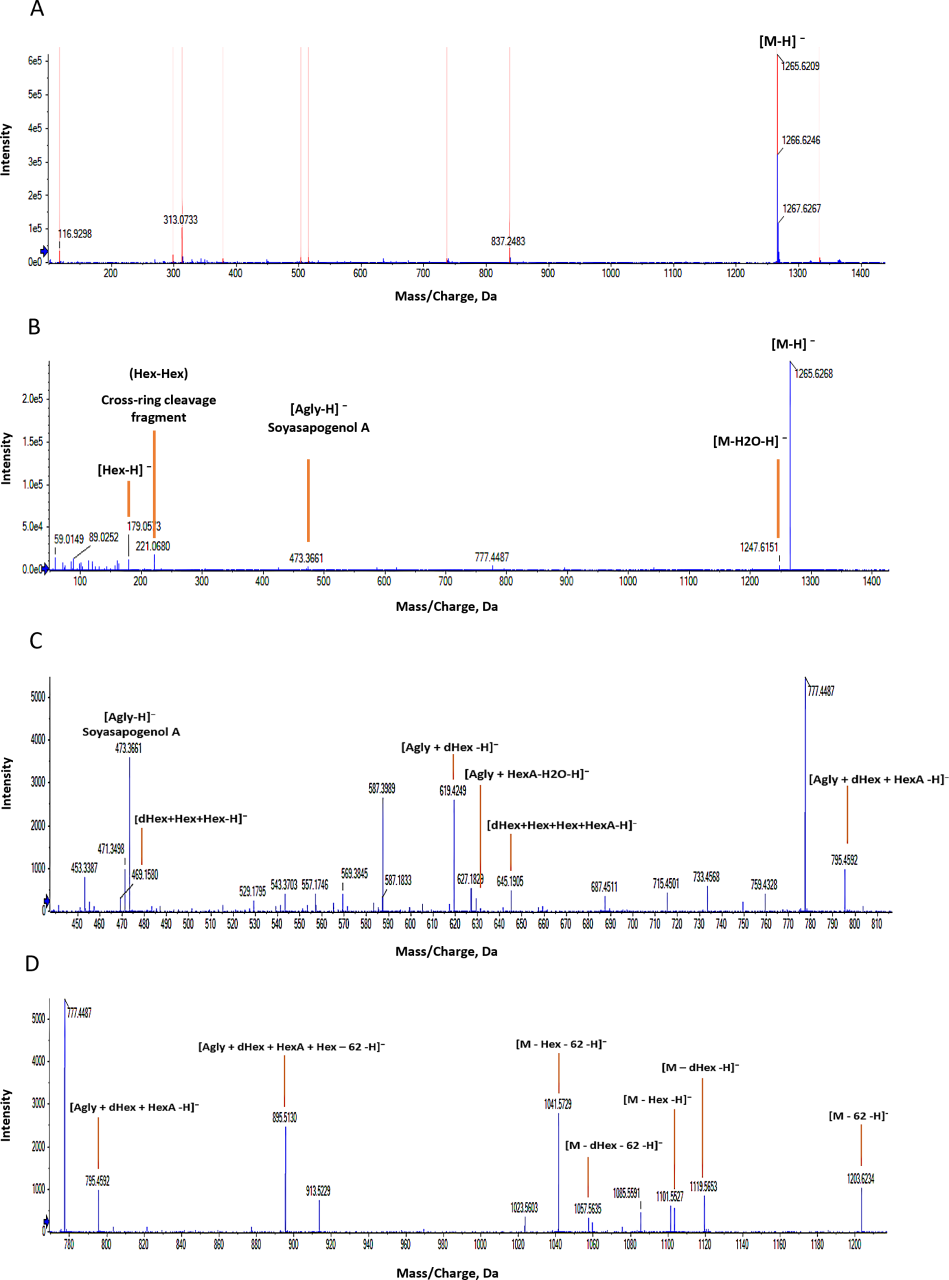
MS/MS spectrum illustrating the fragmentation of the compound (m/z 1265.62) in negative mode, tentatively identified as soyasapogenol A - dHex × HexA-Hex-(Hex-dHex). **(A)** MS spectrum of the compound. **(B)** MS/MS fragmentation of the compound, showing the precursor ion at m/z 1265.62, with the aglycone detected at m/z 473.36. **(C)** Zoom from m/z 450 to 810, and **(D)** Zoom from m/z 780 to 1250, both graphs displaying characteristic fragments that indicate the connectivity of the sugar chains.

In total, 48 saponins were tentatively identified in the harvest residues, a complete list of these compounds, including their mass-to-charge ratios (m/z), retention times (RT), and molecular formulas, is provided in the **Supplementary Material**. **Figure 2** illustrates the identification of a Group A saponin (compound 3) using negative electrospray ionization mass spectrometry (ESI-MS/MS). The precursor ion (M-H)^-^ was detected at m/z 1265.62 (**Figure 2A**). Two fragment ions were observed from the precursor: m/z 1119.56, corresponding to the loss of a deoxyhexose (dHex, −146 Da), and m/z 1103.56, corresponding to the loss of a hexose (Hex, −162 Da). The simultaneous loss of both sugars suggests the presence of either two independent sugar chains or a branched terminal structure. From the aglycone at m/z 473.36 (soyasapogenol A), the addition of 146 (dHex) to m/z 619.42 and an uronic acid (HexA-H_2_O, 158) to m/z 631.38 are observed (**Figures 2B, C**). This confirms the presence of two distinct sugar chains, one initiated by dHex and the other by HexA, attached to the aglycone, this is a bidesmosidic saponin. The fragment at m/z 795.45 corresponds to (Agly + dHex + HexA), addition of 180 Da (Hex + H_2_O) to m/z 957.51 confirms the presence of a second hexose as calculated based on the precursor mass. From 957.51 fragments at +146 and +162 can be observed respectively at m/z 1103.56 and m/z 1119.56, thereby showing that the second, larger sugar chain is branched. Saponins primarily undergo fragmentation of the glycosidic bonds during CID in negative ion mode. However (Pollier et al., 2011), reported that saponins also undergo cross-ring cleavages of the uronic acid residue, characterized by a neutral loss of 62 Da (H_2_O + CO_2_). A series of fragment ions starting at -62 from the precursor (m/z 1203.62, 1057.56, 1041.57, 895.51, 733.45, and 587.39) independently confirms the composition and connectivity of the larger sugar chain (**Figure 2D**).

In the lower mass region of the negative-mode MS/MS spectra, characteristic fragment ions provided additional structural insights into the sugar moieties and their connectivity (**Figure 2B**). Fragment ions at m/z 221.06 and m/z 205.07, attributed to cross-ring cleavages within the saccharide units (Wang et al., 2015), respectively, confirm the presence of two hexoses linked together and a hexose linked to a deoxyhexose. The observation of fragments at m/z 469.15 (Hex + Hex + dHex) confirmed the presence of a deoxyhexose and two hexoses in a linked arrangement, while m/z 645.19 (Hex + Hex + dHex+ HexA) shows that the second sugar chain consists of a single deoxyhexose unit (**Figure 2C**). Further confirmation of the saponin was found in the MS/MS spectra of the precursor ion (M+Na)^+^ at m/z 1289.60 in positive mode (**Figure 1** in the **Supplementary Material**). Fragments at m/z 185.03 (Hex + Na)^+^, m/z 331.09 (Hex + dHex + Na)^+^, m/z 347.09 (Hex + Hex + Na)^+^, m/z 493.14 (Hex2 + dHex + Na)^+^ and m/z 669.17 (Hex2 + dHex+ HexA + Na)^+^, but not at m/z 815 (Hex2 + dHex2+ HexA + Na)^+^ confirm that the second dHex is bound to another hydroxyl-function of the aglycone. Based on the comparison of fragment ions in negative ionization modes, the tentative structure of the saponin is proposed as soyasapogenol A- dHex × HexA-Hex-(Hex-dHex). Following this reasoning, the other 47 saponins were tentatively identified.

The most abundant saponins identified in the harvest residues contain soyasapogenol B (m/z: 457) as aglycone (Kinjo et al., 1994); m/z 941.51 (35; dHex-Hex-HexA-Soyasapogenol B), m/z 795.45 (37; Hex-HexA-Soyasapogenol B) and m/z 633.40 (44; HexA-Soyasapogenol B). Two other group B saponins were identified at m/z 1043.54 (29) and 1011.51 (36). These compounds exhibited distinct fragmentation patterns, with initial neutral losses of 102 (C_4_H_6_O_3_) and m/z 70 (C_3_H_2_O_2_), respectively. Since the same masses are found added to the m/z of the aglycone, it indicates that these currently unknown moieties are attached to the aglycone. Group E, the second most abundant group, contains a prominent molecule identified as soysapogenol E-HexA-Hex-dHex detected at m/z 939.49 (27 and 40), with the big difference in retention time between these two isoforms, one is probably a fragment of a molecule that was not fragmented. In addition to Group B and Group E, 12 compounds from Group A were identified, including six monodesmosidic and eight bidesmosidic saponins. These compounds exhibited varying sugar chain compositions attached to different aglycones, further expanding the diversity of saponins in the extracts. Four molecules belonging to the DDMP group were identified, each containing a DDMP (2,3-dihydro-2,5-dimethyl-4H-pyran-4-one) moiety attached to the aglycone soyasapogenol B. These compounds exhibit variations in their sugar chains.

Additional aglycones were also identified in this study including soyasapogenol C (Arulkumar et al., 2022), soyasapogenol E (Buoso et al., 2022), hederagenin, bayogenin, olean-12-ene-3b4diol, and Kudzusapogenol B. Furthermore, an unknown aglycone, designated Aglycone D, was observed, yielding an anion at m/z 489 with a molecular formula of C30H50O5, a structure not previously described in Lupinus species. Based on the findings of (Pollier et al., 2011), aglycone D may represent an oxidation product of soyasapogenol A.

#### 3.1.2 Identification of flavonoids

**Figure 3:**
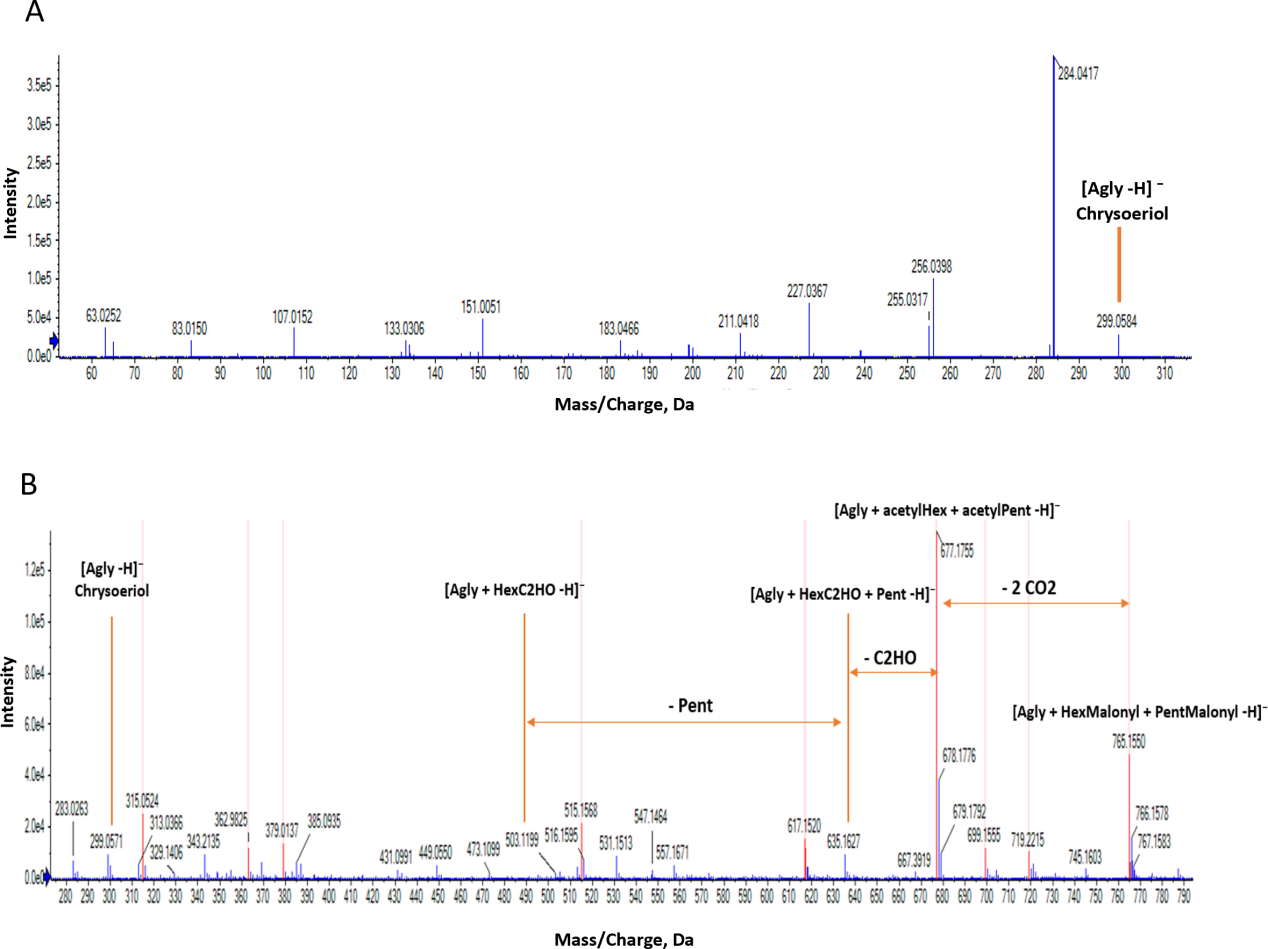
**(A)** MS/MS spectrum illustrating the fragmentation of compound (54) detected in negative mode at m/z 299.05, RT 27.37, and tentatively identified as Chrysoeriol. **(B)** MS spectrum showing the detection of compound (56) (m/z 765.15, RT 25.76), tentatively identified as chrysoeriol-(HexMalonyl)-(PentMalonyl). The in-source fragment observed at m/z 677.17 corresponds to the loss of two CO2 molecules from the malonyl structure, while the fragment at m/z 635.16 represents the molecule lacking a malonyl group and one CO2.

Flavonoids, characterized by their C6–C3–C6 backbone, are major phenolic compounds in legumes and are classified into various subgroups, including anthocyanins, flavones, flavonols, flavanones, flavan-3-ols, and isoflavones. These subgroups differ in their oxidation degree, hydroxylation patterns, and structural variations (Chen et al., 2023). In this study, 80 compounds were tentatively identified and categorized into four subclasses. Among these, 21 flavones were identified, with chrysoeriol and its derivatives being the most abundant. The free aglycone of chrysoeriol was detected at m/z 299.05 (116) (**Figure 3A**), and its identification was confirmed with a standard. Furthermore, 12 chrysoeriol glycosides were identified, six of which contained malonylated sugar moieties.

**Figure 3B** illustrates the MS fragmentation of a dimalonylated glucosylated Chrysoeriol (107). In negative ionization mode, the precursor ion (M-H)^-^ was detected at m/z 765.15, corresponding to Chrysoeriol-(malonylHex)-(malonylPent). A prominent in-source fragment was observed at m/z 677.15, resulting from the loss of 2 molecules of CO_2_ corresponding to 88 Da. MS/MS of this in-source fragment shows losses of 174 and 204, corresponding to acetylPent and acetylHex, respectively. The high intensity of the in-source fragment ion at m/z 677.15 shows that decarboxylation of the malonyl groups is a predominant pathway during negative mode analysis (Galvis Sánchez et al., 2003). This loss of 44 Da was consistently observed across the dataset, in some cases making the malonylated compound of such low intensity that it was not fragmented. The resulting 42 Da remaining attached to the sugar moieties, an acetyl-group, can easily lead to the misinterpretation of the compound as being acetylated. To resolve this ambiguity, the correct mass of the compound can be found from the m/z value in positive mode. This compound was detected in positive mode at m/z 767.16, confirming that the compound is malonylated. Furthermore, an in-source fragment was observed at m/z 549.11, corresponding to chrysoeriol-malonylHex, confirming that both sugars are malonylated and that it is the malonylHex that is attached to the aglycone.

Apigenin (113) (m/z: 269.04), another flavone, was also identified based on the RT and MS/MS of a standard. Similar to the malonylated chrysoeriol derivatives, malonylation of apigenin glycosides was confirmed by the loss of CO_2_ in negative and the exact mass determination in positive. Quercetin, dihydrokaempferol, and their derivatives were identified, along with malonylated glycoside forms of kaempferol, all classified within the flavonol subclass (Ben Hassine et al., 2021; Wojakowska et al., 2013). Additionally, O-methylated flavonols were detected, including isorhamnetin along with its glycosides and malonylated derivatives. Malonyl glycosides were identified for the different isoflavones known in lupins (Wojakowska et al., 2013), these are genistein, hydroxygenistein, and the prenylated luteone and wighteone. For the latter compound, the sulfated form was likewise identified, a modification not found for the other flavonoids. Finally the coumaronochromone lupinalbin A, a known anti-inflammatory but also fungi-toxic agent (Kim et al., 2018; Von Baer et al., 2006) and the main coumarin in different lupine species was also identified, further expanding the diversity of this subclass. Furthermore, within the flavanone subclass, naringenin was identified, adding to the range of flavonoids characterized in this study.

#### 3.1.3 Identification of other chemical classes in the harvest residues of the *Lupinus* species

In addition to saponins and flavonoids, a total of 30 lipids were identified, comprising four subclasses of glycerophospholipids: glycerophosphocholines, glycerophosphoinositols, glycerophosphoethanolamines, and glycerophosphoglycerols. Two octadecanoids, 12,13-dihydroxy-9Z-octadecenoic acid (DiHOME) and 9,10,13-trihydroxy-11E-octadecenoic acid (TriHOME), were also detected. Furthermore, two glycolipids classified as digalactosyldiacylglycerols (DGDG) and monogalactosyldiacylglycerols (MGDG), along with four isoprenoid-derived metabolites, were identified. Ceramides, including glucosylceramides such as soycerebroside II, were tentatively identified based on spectral features. In addition, other bioactive metabolites were identified. Two quinone derivatives, nepodin and emodin-3-O-sulfate, were detected. Other compounds included amino acids such as N-acetylphenylalanine, salicylic acid glycosides, and gallic acid, phenolic acids, including dihydroxybenzoic acid, and organic acids like malic acid were also observed. Phenolic glycosides, such as guaiacylglycerol (8-O-4) ferulic acid hexoside, as well as secondary alcohols like pantothenic acid, were present. Additionally, isocoumarins, including the mycotoxin alternariol (AOH) and its derivatives produced by *Alternaria* fungi, were identified. A complete list of these compounds, including their mass-to-charge ratios (m/z), molecular formulas, retention times (RT), and their class and sub-class is provided in the **Supplementary Materials**.

### 3.2 Comparative metabolite analysis of saponins and flavonoids in harvest residues of Lupinus albus and Lupinus angustifolius

**Figure 4:**
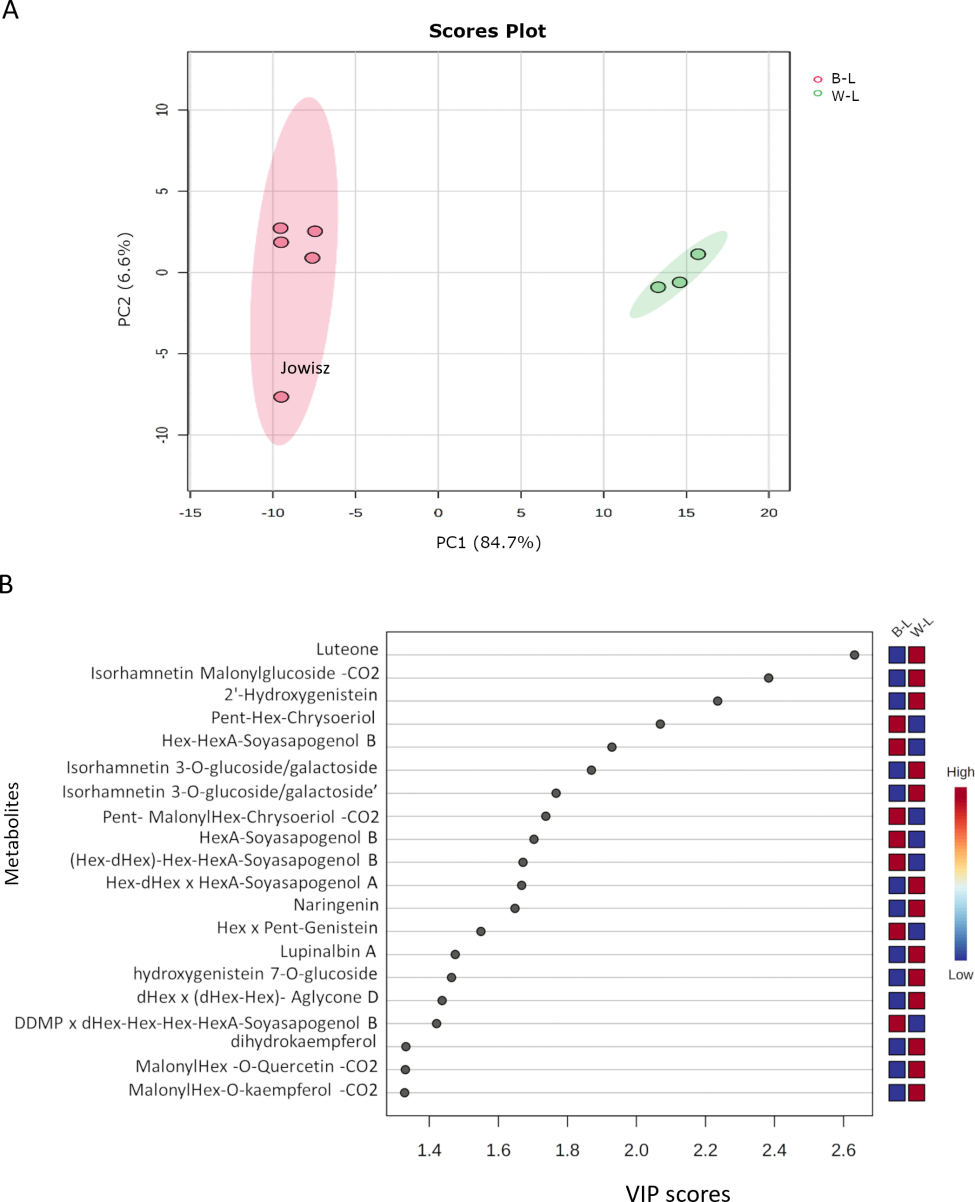
**(A)** PLS-DA scores plot showing the separation between *Lupinus albus* (W-L, green) and *Lupinus angustifolius* (B-L, red) based on the saponin and flavonoid profiles. The first principal component (PC1) explains 84.7% of the variance, while PC2 accounts for 6.6%. **(B)** VIP scores plot highlighting the top 20 metabolites contributing most to species differentiation, based on PLS-DA analysis.

The comparison between *L. albus* and *L. angustifolius* was conducted based on their saponin and flavonoid profiles, as these compounds were the predominant metabolites identified in both species. A total of 127 metabolites (80 flavonoids and 47 saponins) were used for multivariate and univariate statistical analysis. Partial Least Squares Discriminant Analysis (PLS-DA) revealed a clear separation between the two species, indicating distinct metabolic compositions (**Figure 4A**). The first principal component (PC1) accounted for 84.6% of the total variance, representing the major metabolic differences between the species. In contrast, the second principal component (PC2), which accounted for only 6.6% of the variance, primarily reflected minor intra-species variability. The metabolic variability was higher in blue lupins compared to white lupins, with the Jowisz variety exhibiting the greatest variation along PC2. To further identify the key metabolites contributing to species differentiation, Variable Importance in Projection (VIP) analysis was performed.

The 20 metabolites with the highest VIP scores, the main discriminating factors between *L. albus* and *L. angustifolius*, are shown in **Figure 4B**. Among them, 14 belong to the flavonoid class, with luteone emerging as the top-ranking metabolite, exhibiting greater abundance in *L. albus*. The list also features three isoflavones (lupinalbin A, 2’-Hydroxygenistein, and Hex x Pent-Genistein), six flavonols (including three isorhamnetin derivatives, dihydrokaempferol, and three malonylated compounds, with quercetin, chrysoeriol, and kaempferol as aglycones), one flavanone (naringenin), and one flavone (Pent-malonylHex-chrysoeriol-CO_2_). Among saponins, two belong to group B, one to DDMP-group, one to group A, and one contains Aglycone D. Notably, saponins with soyasapogenol B were more abundant in *L. angustifolius*, while the others were higher in *L. albus*.

**Figure 5:**
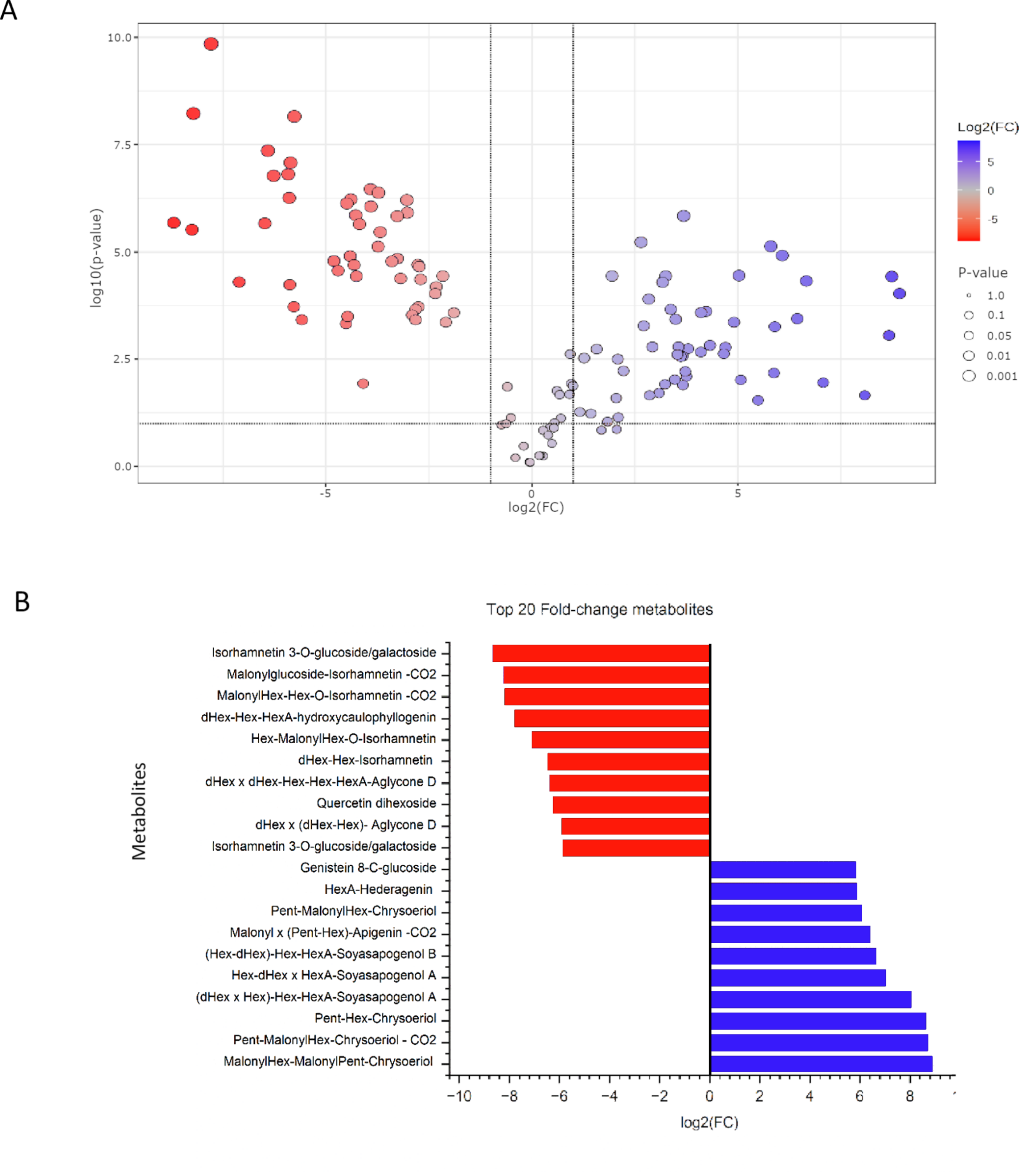
**(A)** Volcano plot illustrating the distribution of metabolites based on fold change (log2FC) and statistical significance (-log_10_(p-value)). Metabolites in red are more abundant in white lupins, while those in blue are more abundant in the blue lupins. Grey points represent metabolites with no significant differences. **(B)** Top 20 Fold-Change Metabolites between *L. albus* and *L. angustifolius* based on log_2_ fold change (log_2_FC) values. red bars represent metabolites that are more abundant in white lupins, while blue bars indicate metabolites more abundant in the blue lupins.

To further validate the metabolic differences identified through multivariate analysis, an univariate statistical analysis was performed. A volcano plot was generated to visualize the distribution of significantly altered metabolites between *L. albus* and *L. angustifolius* (**Figure 5A**). Out of the 127 metabolites, 49 were significantly more abundant in *L. angustifolius*, 50 were significantly more abundant in *L. albus*, and 28 showed no significant variation between the two species.

**Figure 6:**
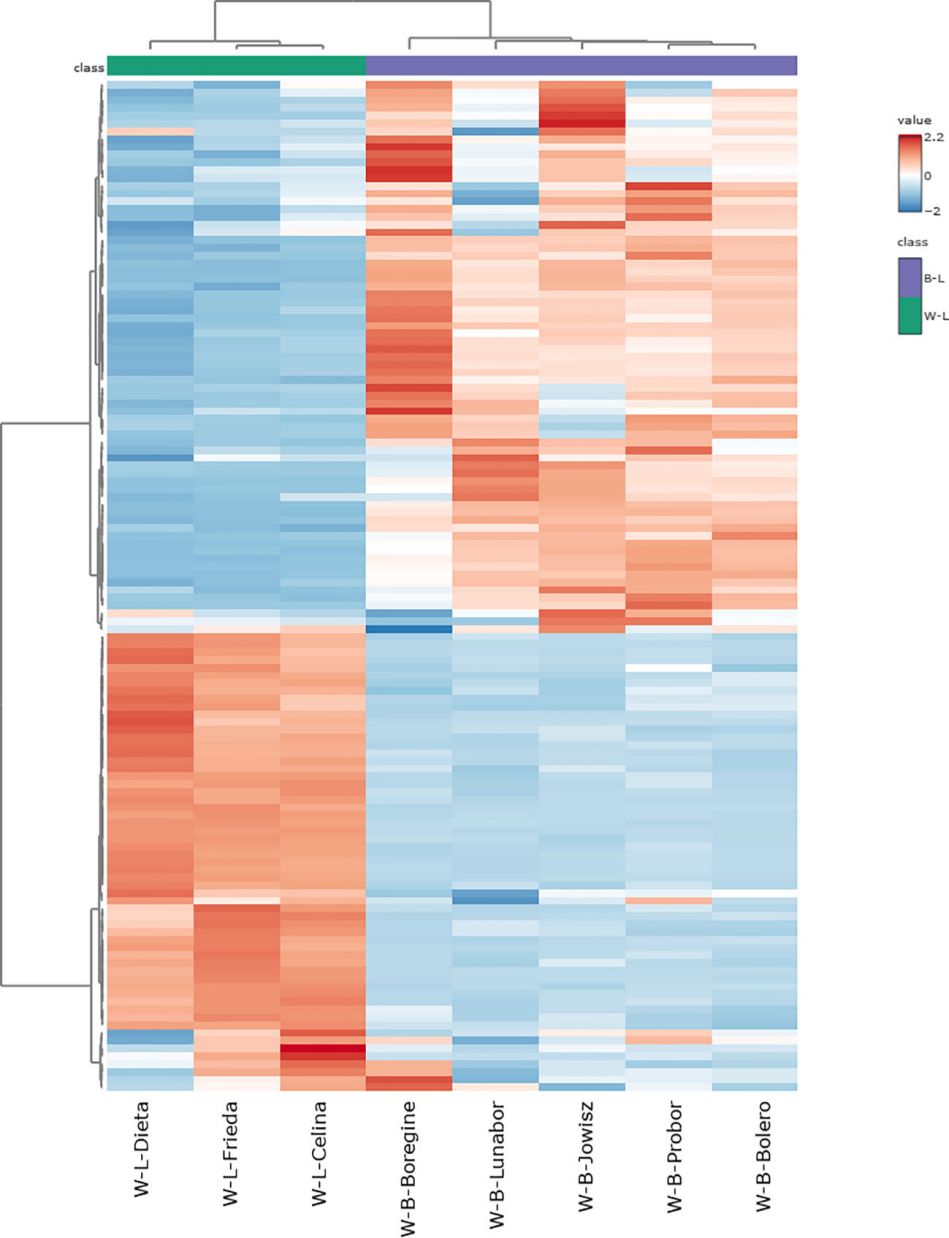
Heatmap of Metabolite Abundance in *L, albus* and *L. angustifolius*. The color scale represents metabolite abundance, with red indicating higher concentrations and blue representing lower concentrations.

Among the significantly altered metabolites, the 10 with the highest fold changes and the 10 with the lowest fold changes are presented in **Figure 5B**. Seven of the ten metabolites with the lowest fold change, those most abundant in *L. angustifolius*, belong to the flavonol subclass, with six being isorhamnetin derivatives. Regarding saponins, two molecules with aglycone D and one with Kudzusapogenol B as aglycone were found in lower abundance in *L. angustifolius.* For the most abundant metabolites in blue lupins, four chrysoeriol derivatives were highly present, along with one apigenin derivative and one genistein derivative. Regarding saponins, two saponins from group A, one from group B, and one saponin with hederagenin as the aglycone were more abundant in *L. angustifolius*. A heatmap (**Figure 6**) was generated to illustrate metabolites distribution in the two species. Hierarchical clustering analysis revealed distinct metabolic profiles, clearly separating the two species. This clustering further confirms the metabolic divergence between the species, aligning with findings from multivariate (PLS-DA, VIP) and univariate (fold change, statistical significance) analyses.

**Figure 7:**
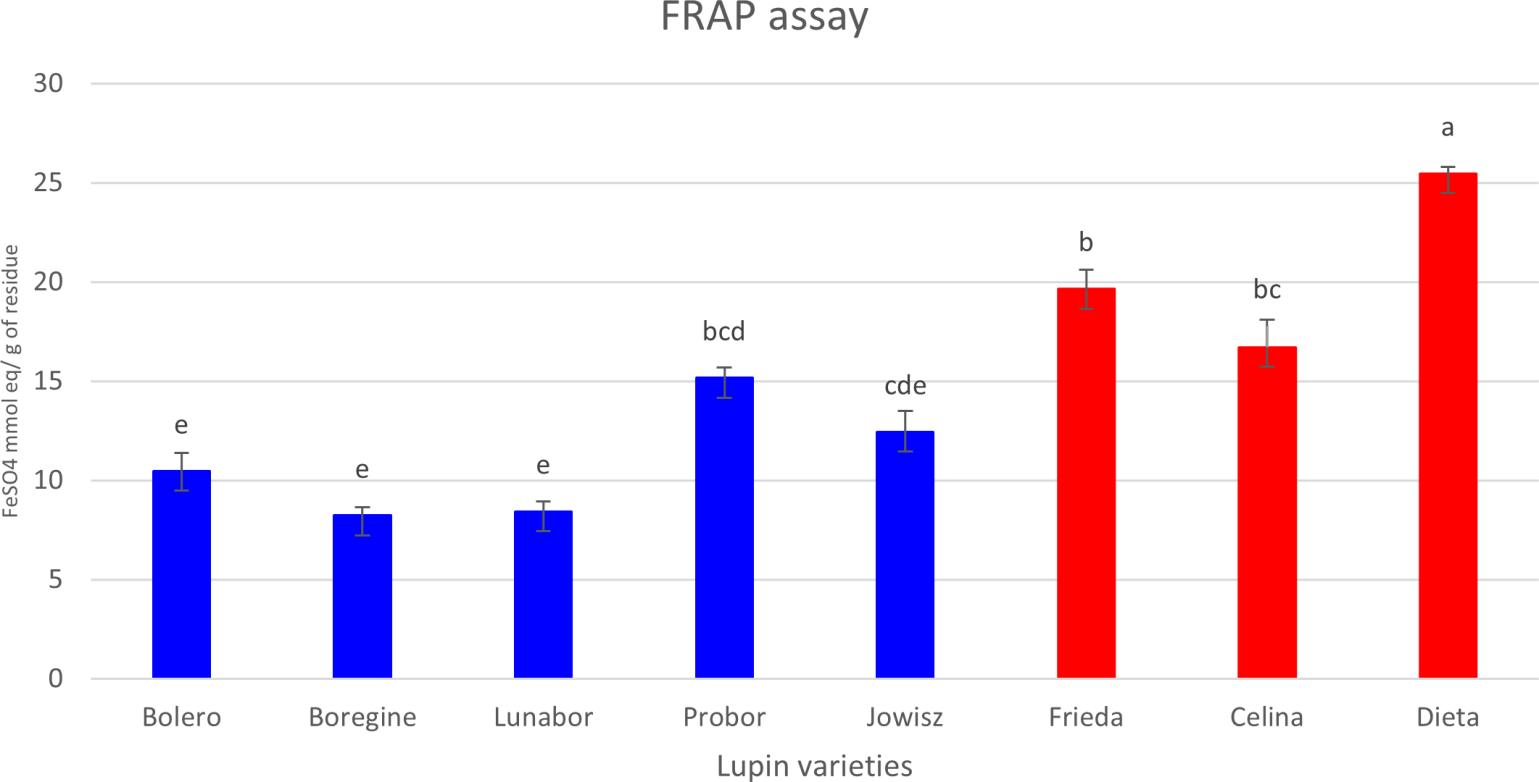
Antioxidant activity (FRAP) of extracts from different Lupinus varieties. Blue bars represent blue lupin (*L. angustifolius*), while red bars represent white lupin (*L. albus*). Statistical differences between groups were analyzed using Tukey’s *post-hoc* test following one-way ANOVA, performed in OriginPro 2024. Data are presented as mean ± standard deviation (SD), with significant differences indicated by different letters (p < 0.05).

The antioxidant activity of the extracts was evaluated using the Ferric Reducing Antioxidant Power (FRAP) assay. The results show that extracts from the three varieties of white lupins exhibited higher antioxidant activity compared to those of the blue lupin varieties (**Figure 7**). The correlation analysis was conducted using the Spearman rank correlation test on normalized metabolite data to evaluate the relationship between metabolite abundance and antioxidant capacity. The results revealed a strong positive correlation between several flavonoids and antioxidant activity, with dihydrokaempferol derivatives displaying the highest correlation values. Dihydrokaempferol malonylHex-CO_2_ (r = 0.976) and dihydrokaempferol O-hexoside (r = 0.976) were the most strongly correlated metabolites, followed by chrysoeriol hexoside (r = 0.952), quercetin dihexoside (r = 0.952), and various isorhamnetin derivatives (r > 0.88). These results indicate that flavonoids, particularly those with dihydrokaempferol, isorhamnetin, quercetin, and naringenin as aglycone, may play a central role in determining the antioxidant activity of extracts made from the two species. Conversely, a strong negative correlation was observed for saponins, suggesting an inverse relationship with antioxidant activity. HexA-Soyasapogenol A, HexA-Soyasapogenol E, and HexA-Hederagenin exhibited the strongest negative correlations (r = -0.976), alongside other saponins such as Hex-HexA-Soyasapogenol B (r = -0.928) and HexA-Soyasapogenol B (r = -0.904). In addition, some wighteone, luteone, and chrysoeriol derivatives also showed strong negative correlations (r < -0.9). These findings suggest that while flavonoids contribute significantly to antioxidant capacity, saponins do not appear to enhance antioxidant activity and may even be inversely associated with radical scavenging potential.

## 4 Discussion

The increasing global demand for plant-based protein has led to a growing interest in the cultivation of *Lupinus albus* and *Lupinus angustifolius*, two economically significant legume species native to the Mediterranean region (Lamrabet et al., 2022). Given their agricultural and nutritional importance, numerous studies have investigated the metabolic composition of their seeds, roots, and aerial parts, consistently identifying phenolic compounds, alkaloids, tannins, and fatty acids as the predominant metabolite classes (Ben Hassine et al., 2021; Dunshea et al., 2001; Mazumder et al., 2024). In the present study, we analyzed the metabolic composition of the harvest residues of *L. albus* and *L. angustifolius* and compared their phytochemical profiles, thereby exploring the potential to valorize this abundant agricultural residue. Methanolic extracts of the harvest residues of different varieties of the two species were subjected to HPLC-MS/MS analysis, leading to the tentative identification of 181 compounds spanning multiple phytochemical classes.

Untargeted metabolomic profiling revealed the predominance of two major phytochemical classes: saponins and flavonoids. While flavonoids and isoflavonoids profiles of *L. albus* and *L. angustifolius* have been previously reported (Bednarek et al., 2003; Ferchichi et al., 2021), to the best of our knowledge, no prior study has identified saponins as a major class in these species. In the present study, 47 saponins were identified in the harvest residues, primarily belonging to the soyasaponin subclass, a group of oleanane-type triterpenoid glycosides that have been previously reported in lupins (Bulut et al., 2023; Gunning et al., 2013; Ruiz et al., 1995). These saponins were categorized into four major groups: A, B, E, and DDMP soyasaponins, with notable differences in their distribution between *L. albus* and *L. angustifolius*. The most abundant saponin detected in both species was soyasapogenol I, a group B soyasaponin, identified at m/z 941 (35). This compound was significantly more abundant in *L. angustifolius*. Earlier studies have confirmed the presence of soyasapogenol I in *L. angustifolius*, supporting our findings for this species. However, no prior reports have documented its presence in *L. albus* (Ruiz et al., 1995; Woldemichael and Wink, 2002). Additionally, soyasapogenol I have been identified in other legumes, including lentils, green peas, and soybeans (Rupasinghe et al., 2003; Sagratini et al., 2013) and has been associated with several bioactive properties, including cholesterol-lowering effects (Lee et al., 2005). Anticarcinogenic and anti-inflammatory activity (Guang et al., 2014; Lee et al., 2010).

The second most abundant group of soyasaponins was group E, with a major compound identified at m/z 939 (27, 40). These compounds are photo-oxidation products of group B soyasaponins and, according to the literature, are often considered artifacts rather than naturally occurring metabolites (Bianco et al., 2018; Rupasinghe et al., 2003). Similar to group B soyasaponins, group E was also found in higher abundance in *L. angustifolius*, suggesting a species-dependent metabolic transformation or increased oxidative susceptibility. A subclass derived from group B soyasaponins is the DDMP group. These compounds represent the predominant genuine soyasapogenol B-derived soyasaponins, characterized by their conjugation at position 22 with a DDMP moiety (Bianco et al., 2018). DDMP saponins have been associated with notable health benefits (Sayama et al., 2012), including radical scavenging properties, anti-mutagenic activity (Berhow et al., 2002), and the prevention of colon cancer proliferation (Tsai et al., 2010). Several DDMP-conjugated saponins were identified, with a higher abundance in *L. angustifolius*, following the distribution pattern observed for group B and E saponins. Group A soyasaponins, which are known to contribute to the bitterness and aftertaste of legume seeds (Rupasinghe et al., 2003), were also detected. These molecules are typically found in soybeans, where they undergo acetylation at the sugar moieties through the action of specific O-acetyltransferases (Ma and Shang, 2023) However, in this study, group A saponins were identified in both *L. albus* and *L. angustifolius*, but notably, they were not acetylated. This lack of acetylation may be attributed to the absence or reduced activity of O-acetyltransferase enzymes responsible for this modification in *Lupinus* species. Moreover, these compounds were found in higher abundance in *L. angustifolius*. The results of this study indicate that soyasaponins were more abundant in the harvest residues of *L. angustifolius* than in *L. albus*, contributing to the metabolic differentiation between the two species. This difference suggests species-specific variations in triterpenoid biosynthesis, regulatory mechanisms, or metabolite accumulation.

Beyond the predominant soyasaponin groups, several additional triterpenoid aglycones were identified for the first time in both species, expanding the known phytochemical diversity of *Lupinus*. Among them, olean-12-ene-3β,24-diol (24-hydroxy-β-amyrin), an intermediate leading to the biosynthesis of soyasapogenol B in legumes by a hydroxylation at the C-22 position (Lambert et al., 2011), was identified with significant abundance in *L. angustifolius*. This compound has been previously reported in members of the *Leguminosae* family (Nascimento et al., 2019), but its presence in *Lupinus* species was not documented before. Hederagenin was also detected, a class of bioactive compounds with well-documented pharmacological properties (Huang et al., 2023). Hederagenin was significantly more abundant in *L. angustifolius*, whereas its derivatives, bayogenin, and the Kudzusapogenol B, were found in significantly higher concentrations in *L. albus*. This species-dependent variation suggests that *L. albus* may possess more active oxidation enzymatic pathways involved in the hydroxylation of hederagenin, potentially leading to the preferential accumulation of these derivatives. Additionally, Aglycone D, an unknown triterpenoid previously described by (Pollier et al., 2011), was also identified. Interestingly, two saponins containing this aglycone were detected exclusively in *L. albu*s. Since aglycone D was originally reported in *Medicago*, this finding supports the hypothesis of conserved triterpenoid biosynthetic pathways among leguminous plants while also revealing distinct species-specific regulatory mechanisms in *Lupinus*.

The harvest residues of both lupin species were characterized by the presence of long and short-chain saponins, including mono- and bidesmosides. The first sugar unit attached to the aglycone was a uronic acid in 97% of the cases, regardless of the aglycone type. The second sugar was typically a hexose or pentose, while the terminal sugar was usually a deoxyhexose. In some cases, the terminal sugar unit was branched, forming a short extension consisting of a hexose and a deoxyhexose. Regarding the differentiation between *L. albus* and *L. angustifolius*, no species-specific glycosylation patterns were observed.

Flavonoid and isoflavonoid glycosides are well-documented in *L. albus* and *L. angustifolius* (Ferchichi et al., 2021). In the present study, 80 compounds were tentatively identified and classified into four subclasses: isoflavonoids, flavones, flavonols, and flavanones. All these subclasses have been previously characterized in roots, leaves, and seeds of the two species (Ben Hassine et al., 2021; Ferchichi et al., 2021; Von Baer et al., 2006). The flavonoid/isoflavonoid profiles of the two species exhibited distinct differences, luteone was identified as the key isoflavone differentiating *L. albus* from *L. angustifolius* (**Figure 5B**). The free aglycone and its glycoconjugates (some of which were malonylated) were found in a significantly higher abundance in *L. albus*. Luteone is the 6-isopentenyl-derivative of 2’-hydroxygenistein, which was also significantly more abundant in *L. albus*. These molecules have demonstrated antimicrobial activity *in vitro* (Harborne et al., 1976; Tahara et al., 1984) and are believed to play a dual role in plant defense. On the one hand, they are secreted constitutively on the surface of healthy plants, where they function as phytoanticipins preformed antimicrobial compounds that provide passive defense against pathogens (VanEtten et al., 1994). On the other hand, evidence indicates that lupin isoflavones, including luteone, may also act as phytoalexins, compounds synthesized in response to pathogen attack, constituting an active defense mechanism (Bednarek et al., 2001). The high abundance of luteone and its derivatives in *L. albus* may indicate a species-specific enhanced resistance to microbial infections. This claim is supported by the lack of detection of fungal toxins in extracts from the harvest residues of *L. albus.* In the extracts from *L. angustifolius* alternariol, a mycotoxin associated with *Alternaria alternata* infection, was identified. This suggests that the residues of *L. albus* were not or less affected by fungal infection, potentially attributed to the higher abundance of 2’-hydroxygenistein, luteone and their derivatives. These isoflavonoids were previously reported in roots and leaves of seedlings in both species, but in much lower concentrations compared to other isoflavonoids (Wojakowska et al., 2013). The high intensity of these compounds in the residues of *L. albus* may be explained by the fact that these compounds are predominantly synthesized and secreted in mature plants. The absence or low concentration of these compounds in *L. angustifolius* could be attributed to the detoxification of phytoalexins by fungal pathogens, a strategy observed in host-specific interactions (Pedras and Ahiahonu, 2005). Additionally, the response of *L. angustifolius* to infection appears to be age-dependent, with young leaves synthesizing luteone as a phytoalexin, while older leaves exhibit a reduced response (Muth et al., 2009). Since this study focuses on harvest residues, which primarily consist of mature plant material, this may explain the lower abundance of these compounds in *L. angustifolius*.

The isoflavonoids, wighteone and genistein, were more abundant in *L. angustifolius* than in *L. albus*. The presence of these natural products with known phytoalexin activity was previously reported (Stobiecki et al., 2010) and, contrary to 2’-hydroxygenistein derivatives, they typically accumulate in response to pathogen infection rather than being constitutively produced. The higher abundance of 2’-hydroxygenistein derivatives in *Lupinus albus* compared to *Lupinus angustifolius* suggests that hydroxylation mechanisms may be more active in the white lupin species. This could be attributed to higher expression or activity of flavonoid 2’-hydroxylases in *L. albus* (e.g., CYP81E or related CYP450 enzymes) in *L. albus* (Rasmussen, 2009). Previous studies have demonstrated that wighteone synthesis is primarily induced in older leaves of *L. angustifolius* following pathogen exposure (Muth et al., 2009). Similarly, genistein production was shown to increase in later stages of plant development upon infection with *Colletotrichum lupini* (Wojakowska et al., 2013). The higher abundance of these compounds in *L. angustifolius* suggests a species-specific defense strategy, potentially linked to pathogen-induced stress responses. A coumaronochromone isoflavonoid that was significantly more abundant in *L. albus* was lupinalbin A. This compound was previously identified in germinated seeds of *L. albus* (Shams Eldin et al., 2023) and has been shown to possess aryl hydrocarbon receptor (AhR) agonistic activity (Ateba et al., 2014), highlighting its potential biological significance.

The flavones apigenin and chrysoeriol (3′-O-methylluteolin), along with their glycoconjugated and malonyl derivatives, were detected with higher MS-signal intensities in *L. angustifolius*, consistent with previous reports on the flavonoid profiles of seedlings (Bednarek et al., 2003; Muth et al., 2009). Chrysoeriol and apigenin possess antioxidant, anti-inflammatory, cytotoxic, and hypoglycemic properties (Chen et al., 2012). Additionally, two flavones not previously identified in *Lupinus* species were detected in this study: tricin glucoside and hispidulin glucoside. Tricin was significantly more abundant in *L. angustifolius*, whereas no significant difference in hispidulin levels was observed between the two species. These flavones have already been reported in other legumes, including kidney bean, cowpea, fava bean, soybean, and pea (Zhao et al., 2024), and are known for their various bioactive properties (Chaudhry et al., 2024; Lam et al., 2021).

Concerning the other identified molecules, salicylic acid (SA) and its glucoside form, along with dihydroxybenzoic acid and its derivative, were found in higher abundance in *L. albus* compared to *L. angustifolius*. SA plays a crucial role in plant innate immunity, acting as a key regulator of defense responses (Yan and Dong, 2014). In addition, biochemical assays have shown that glycosylated dihydroxybenzoic acid is a major catabolite of SA, as it undergoes glycosylation to form glucose and xylose conjugates both *in vitro* and *in vivo*, explaining the observed positive correlation between these metabolites (Huang et al., 2018). Glucosylated gibberellins, pivotal compounds in the regulation of plant growth and development (Castro-Camba et al., 2022), were detected with significantly higher intensities in *L. albus*. Previous studies have demonstrated that certain gibberellin glucosides act as immunoreactive substances (Hasegawa et al., 1994), potentially playing a role in plant defense mechanisms. Combined with the flavonoid data, these findings suggest that *L. albus* may be less susceptible to pathogens compared to *L. angustifolius*. Comparative studies highlighted differences in pathogen resistance strategies between the two species. The genetic analyses have discovered several loci in *L. albus* that are associated with anthracnose resistance, thus supporting the existence of a polygenic resistance mechanism that makes the species more resistant to pathogen attack (Alkemade et al., 2022). On the other hand, *L. angustifolius* relies mainly on a single dominant resistance gene, which provides a high level of resistance, but is less durable due to pathogen adaptation and the development of resistance (Książkiewicz et al., 2021). These differences are not only related to pathogen defense, but also correspond to more global allocation patterns of resources when stressed. During biotic stress, *L. angustifolius* maintains growth and yield, while the growth of *L. albus* is more impacted due to the allocation of more resources to increase the concentration of defensive metabolites (del Pilar Vilariño & Ravetta, 2008). However, although *L. albus* appears to possess a more complex immune response, resistance cannot be generalized at the species level. Ecologically, these species-specific differences in metabolite accumulation and defense strategies may also reflect an evolutionary adaptation to their original habitats and pathogen pressures. Even when grown under the same conditions, these evolved traits influence how each species interacts with its environment, affecting their resilience and ability to thrive in different environments. Moreover, pathogen susceptibility varies based on genetic diversity within each genotype, environmental factors, and the extent of disease pressure.

Musizin/nepodin glucoside, an anthraquinone, was tentatively identified as the main phenolic compound in *L. albus*. To our knowledge, this compound has never been reported in lupins but was identified in other *Fabaceae* (Abdellatif et al., 2023; Santana et al., 2015). Anthraquinones are known for their diverse bioactive properties, including anticancer and antimicrobial activity (Berillo et al., 2022; Sun et al., 2020), suggesting potential pharmacological applications for the extract of the harvest residues of *L. albus*. However, further functional validation through *in vitro* assays is necessary to confirm their efficacy for practical applications.

Interestingly, no alkaloids were detected in the harvest residues of either lupin species. This contrasts with previous reports that analyzed harvest residues of the two species using gas chromatography (GC), indicating that, although alkaloid concentrations in stems, leaves, and pods generally decline as they accumulate in seeds, *L. albus* retains considerable amounts of alkaloids in its straw and pod shells (Maia et al., 2023). The observed variation may be attributed to the selective breeding of the genotypes investigated in this study for low alkaloid content, as these compounds are considered antinutritional factors (Enneking and Wink, 2000). However, another important factor is the methodology used. The extraction protocol used in this study was selected to target polar compounds such as flavonoids and saponins but is not ideal for isolating alkaloids. In most cases, alkaloid extraction requires the use of acidic solvents such as trichloroacetic acid. Furthermore, we used UHPLC for analysis, which is well-suited for non-volatile compounds, whereas alkaloids are often better detected with gas chromatography. This means the lack of detected alkaloids likely reflects both the extraction method and the analytical approach, rather than their actual absence in the plant residues. Future work using targeted extraction and GC-MS analysis could help confirm the presence or absence of alkaloids and complete the phytochemical profile.

The antioxidant capacity of *Lupinus albus* extracts was significantly higher than that of extracts from *L. angustifolius*, likely due to the higher abundance of flavonols, including dihydrokaempferol, isorhamnetin, and quercetin, as well as flavanones, particularly naringenin, which are well known for their radical-scavenging activity (Cavia-Saiz et al., 2010; Jan et al., 2022; Seyoum et al., 2006). Previous studies have reported a positive correlation between flavonoid content and antioxidant capacity in *L. albus* (Grela et al., 2017). Additionally, phenolic acids such as protocatechuic acid and *p*-hydroxybenzoic acid have also been linked to the antioxidant potential of *L. albus* (Siger et al., 2012). However, the results of this study contrast with those reported for methanolic extracts of *L. albus* and *L. angustifolius* seeds, where *L. angustifolius* exhibited higher antioxidant capacity (Siger et al., 2012). These differences may be attributed to differences in tissue-specific metabolite composition, as seed constituents differ significantly from those present in harvest residues. Another possible explanation lies in the role of saponins, which were more abundant in the harvest residue of *L. angustifolius* and exhibited a strong negative correlation with antioxidant activity. Saponins, particularly soyasapogenol derivatives, have been associated with various bioactivities, including antioxidant capacity, with the DDMP group being especially known for its antioxidant properties (Yoshiki and Okubo, 1995). However, in this study, the saponins present in the harvest residues appear to contribute minimally to the overall antioxidant potential. Their higher presence in *L. angustifolius* may dilute the effect of antioxidant compounds, further explaining the observed differences between species. The higher antioxidant activity observed in *L. albus* harvest residues suggests a greater capacity for counteracting oxidative stress, which could contribute to enhanced pathogen resistance and environmental adaptability. These findings underscore the potential valorization of *L. albus* residues as a rich source of bioactive compounds

## 5 Conclusion

This study highlights the metabolomic differentiation between *L. albus* and *L. angustifolius* harvest residues, revealing distinct biochemical pathways that define their functional properties. The reported data complement knowledge on species-specific metabolic regulation obtained from other organs. Extracts from the harvest residues of *L. albus* are richer in antioxidant and defense-related metabolites such as flavonols, including dihydrokaempferol, isorhamnetin, and quercetin, as well as flavanones, particularly naringenin, while those of *L. angustifolius* varieties contain higher levels of saponins, especially the soyasaponins, and isoflavonoids like wighteone and genistein. The valorization of these residues may offer sustainable upcycling opportunities by the development of high-value bioproducts. Integrating these bioactive compounds into circular economy models could enhance resource efficiency and promote greener alternatives in food, health, and agriculture sectors. Further research should focus on investigating the bioactivity of these compounds to better understand their mechanisms of action and their industrial potential.

## Data Availability

The original contributions presented in the study are included in the article/**Supplementary Material**. Further inquiries can be directed to the corresponding author.
