# Supplementary material for: Comparative metabolomic profiling of Lupinus albus and L. angustifolius harvest residues: exploring chemical diversity and valorization potential
Source: Front Plant Sci. 2025 Jul 7;16:1617634. doi: 10.3389/fpls.2025.1617634 (PMC12277371; doi:10.3389/fpls.2025.1617634)
Supplement: Supplementary file 1 [file DataSheet1.docx]

**
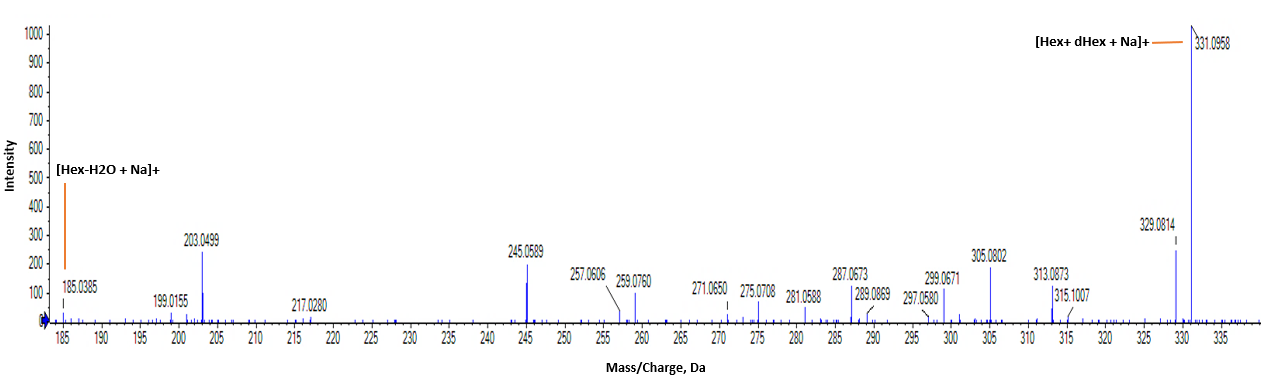
**
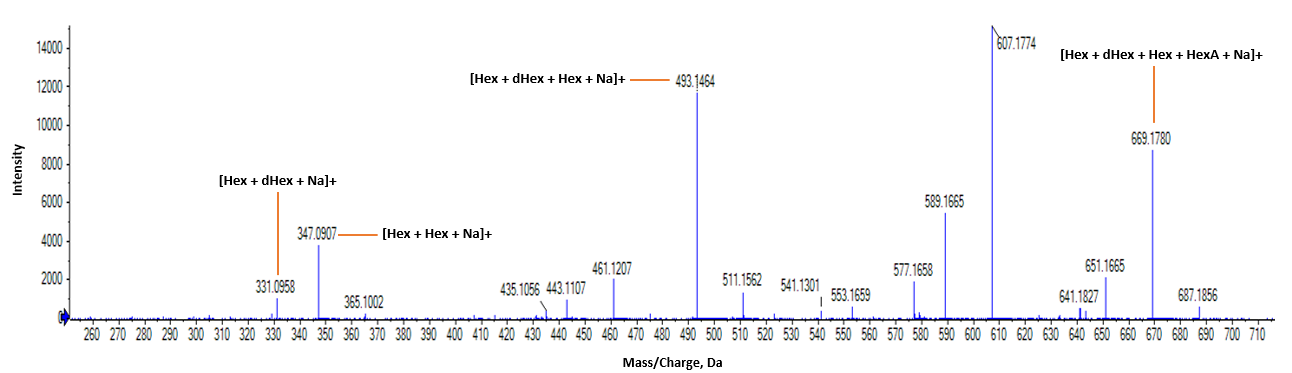
**
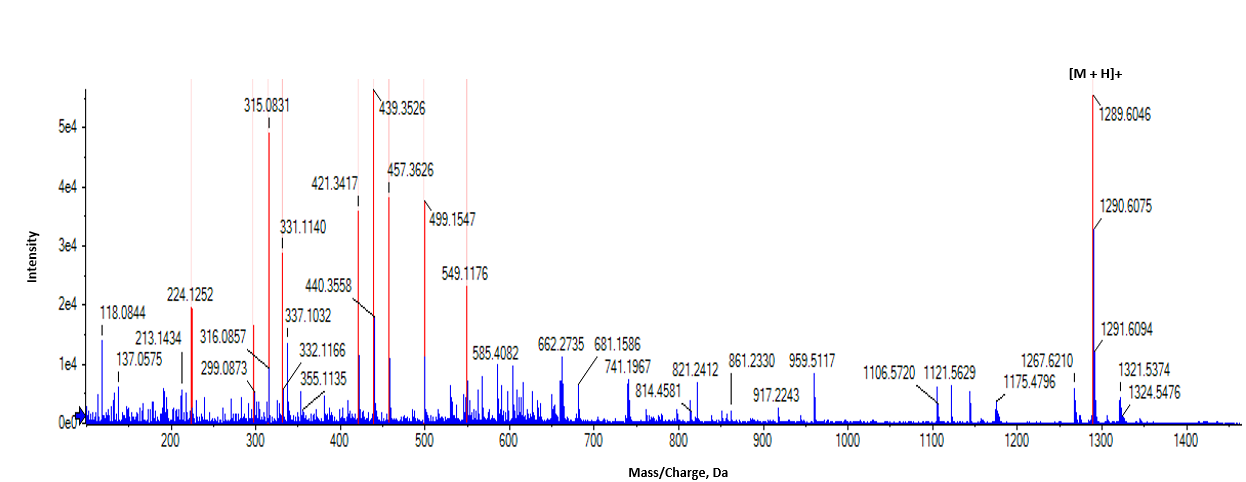
Supplementary material**

B

C

A

**Figure 1:** *MS/MS spectrum illustrating the fragmentation of the compound (m/z 1265.62) in positive mode, tentatively identified as soyasapogenol A - dHex × HexA-Hex-(Hex-dHex). (A) MS spectrum of the compound in positive mode. (B) and (C) MS/MS fragmentation of the compound in positive mode, showing the fragments at m/z 185.03 (Hex + Na)⁺, m/z 331.09 (Hex + dHex + Na)⁺, m/z 347.09 (Hex + Hex + Na)⁺, m/z 493.14 (Hex₂ + dHex + Na)⁺, and m/z 669.17 (Hex₂ + dHex + HexA + Na)⁺, which confirm the chain (HexA-Hex-(Hex-dHex)).*
